# A Practical Treatise on Diseases of the Eye

**Published:** 1877-06

**Authors:** 


### Books Reviewed.

---

*A Practical Treatise on Diseases of the Eye.* By ROBERT BRUDENELL CARTER, F. R. C. S., Ophthalmic Surgeon to St. George's Hospital, etc. etc. With One Hundred and Twenty-four Illustrations. Edited with Additions and Test-Types by John Green, M. D. Philadelphia: Henry C. Lea, 1876, pp. 498.

This is a much handsomer volume than the English edition. It was written for the profession rather than for specialists, and the author says, "Contains but slight reference to modes of practice of which I am unable to speak from experience."

The opening chapter upon the "Anatomy and Physiology of the Eye," is followed by "Examination of the Eye," and the "Ophthalmoscope and its Application."

Two excellent chapters are given to "Principles of Ophthalmie Therapeutics," and "Principles of Ophthalmic Surgery." Special abnormal conditions are considered, beginning with diseases of the eyelids, Conjunctiva, cornea, iris, crystalline lens and its capsule are taken up in the order named. Glaucoma is the subject of a brief chapter, and is invariably treated, by the author, by iridectomy.

Diseases of the fundus oculi receive their full share of attention. It is a pleasure to chronicle a genuine, and heretofore unknown power over "the ills that flesh is heir to." We have one here in the management of symblepharon. "Until recently it was considered to be irremediable and many plans to prevent the union or to prevent the reunion when divided were tried and failed. It was reserved for Mr. T. Pudgin Teale who receives from the Yorkshire iron works large numbers of such cases, to discover the method of treating them and his discovery like most others which are good for anything, has no higher merit than its simplicity. He separated the adherent lid from the eye-ball by careful dissection, and then covered the raw surface of the eyeball by conjunctiva, transplanted from above the cornea, and brought down, sometimes in two flaps, sometimes as an undivided band, adherent at both extremities.

The transplanted conjunctiva adhered readily in its new position, the raw surface of the lower lid had no longer a raw surface opposed to it, and the gap under the upper lid was soon filled up by a new formation, hardly distinguishable from the old." The remainder of the volume is devoted to affections of the ocular muscles, and the "Uses and Selection of Spectacles." It is eminently a book for the times, clear, practical, decided, stating what is the exact *truth*, without hesitation or reservation.

Dr. Green the editor finds many points of difference between himself and Dr. Carter whose practice does not entirely accord with that of other leading ophthalmologists, still in themain he is fully up to the times, and advocates intelligent, safe methods, rather than those that are brilliant, but hazardous and uncertain.

